# Crystal structure and Hirshfeld surface analysis of 3-benzoyl-6-(1,3-dioxo-1-phenyl­butan-2-yl)-2-hy­droxy-2-methyl-4-phenyl­cyclo­hexane-1,1-dicarbo­nitrile

**DOI:** 10.1107/S2056989022004777

**Published:** 2022-05-13

**Authors:** Farid N. Naghiyev, Victor N. Khrustalev, Ekaterina V. Dobrokhotova, Mehmet Akkurt, Ali N. Khalilov, Ajaya Bhattarai, İbrahim G. Mamedov

**Affiliations:** a Department of Chemistry, Baku State University, Z. Khalilov str. 23, Az 1148, Baku, Azerbaijan; b Peoples’ Friendship University of Russia (RUDN University), Miklukho-Maklay St. 6, Moscow 117198, Russian Federation; c N. D. Zelinsky Institute of Organic Chemistry RAS, Leninsky Prosp. 47, Moscow 119991, Russian Federation; d Department of Physics, Faculty of Sciences, Erciyes University, 38039 Kayseri, Turkey; e ‘Composite Materials’ Scientific Research Center, Azerbaijan State Economic University (UNEC), H. Aliyev str. 135, Az 1063, Baku, Azerbaijan; f Department of Chemistry, M.M.A.M.C. (Tribhuvan University), Biratnagar, Nepal; Vienna University of Technology, Austria

**Keywords:** crystal structure, cyclo­hexane conformation, hydrogen bond, van der Waals forces, Hirshfeld surface analysis

## Abstract

The central cyclo­hexane ring of the title compound, C_32_H_28_N_2_O_4_, adopts a chair conformation, with puckering parameters *Q*_T_ = 0.618 (2) Å, θ = 176.72 (19)° and φ = 290 (3)°. In the crystal, mol­ecules are linked by O—H⋯O, C—H⋯O and C—H⋯N hydrogen bonds, forming layers parallel to (100). These layers are linked by weak C—H⋯π inter­actions and van der Waals forces. A Hirshfeld surface analysis indicates that the contributions from the most prevalent inter­actions are H⋯H (41.2% contribution), C⋯H/H⋯C (20.3%), O⋯H/H⋯O (17.8%) and N⋯H/H⋯N (10.6%).

## Abstract 2

In the crystal, O—H⋯O, C—H⋯O, and C—H⋯N hydrogen bonds connect mol­ecules, generating mol­ecular layers parallel to (100). These layers are linked together by weak C—H⋯π inter­actions and van der Waals forces.

## 1. Chemical context

Functionalized derivatives of carbo- and heterocyclic compounds are of great inter­est in the fields of organic synthesis, catalysis, materials science and medicinal chemistry (Zubkov *et al.*, 2018 ▸; Shikhaliyev *et al.*, 2019 ▸; Viswanathan *et al.*, 2019 ▸; Gurbanov *et al.*, 2020 ▸; Khalilov *et al.*, 2021 ▸). In particular, β-dicarbonyl compounds are important chemical substrates for the construction of various classes of organic compounds (Kaur *et al.*, 2021 ▸). 
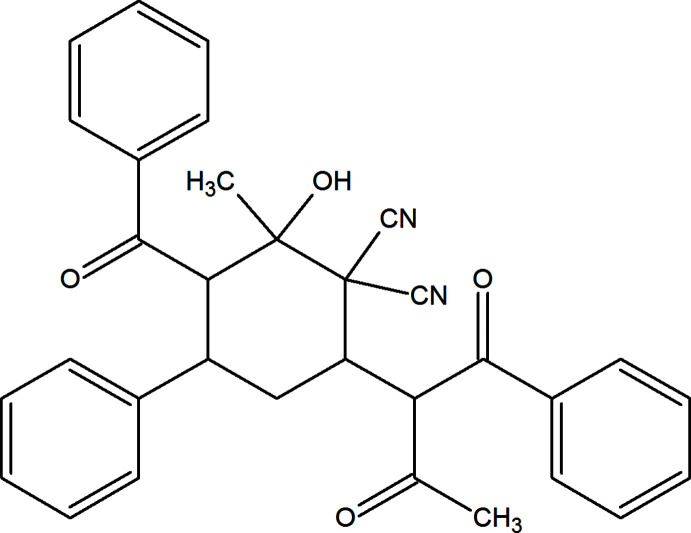


To the best of our knowledge, the inter­action of β-dicarbonyl compounds with phen­yl–allyl­idene–malono­nitriles leads to the formation of xanthene, benzo[*b*]pyran and pyridine derivatives (Bardasov *et al.*, 2014 ▸; Amoozadeh *et al.*, 2018 ▸). Inter­estingly, we discovered that in case of the reaction of one equivalent of phen­yl–allyl­idene–malono­nitrile with two equivalents of benzoyl­acetone at room temperature, a substituted cyclo­hexane derivative was the product. In the context of ongoing structural studies (Safavora *et al.*, 2019 ▸; Aliyeva *et al.*, 2011 ▸; Mamedov *et al.*, 2022 ▸), we report here the crystal structure and Hirshfeld surface analysis of the title compound, 3-benzoyl-6-(1,3-dioxo-1-phenyl­butan-2-yl)-2-hy­droxy-2-methyl-4-phenyl­cyclo­hexane-1,1-dicarbo­nitrile.

## 2. Structural commentary

**Figure 1:**
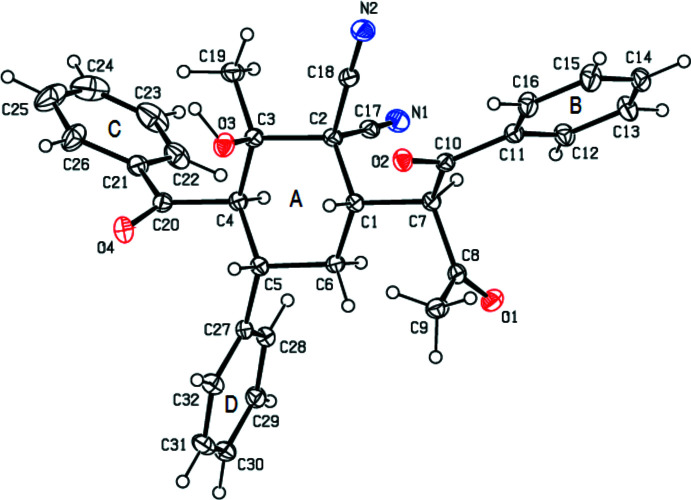
The mol­ecular structure of the title compound, showing the labelling scheme and displacement ellipsoids drawn at the 30% probability level.

In the title compound (Fig. 1 ▸), the central cyclo­hexane ring (*A*: atoms C1–C6) adopts a chair conformation, with puckering parameters (Cremer & Pople, 1975 ▸) *Q*_T_ = 0.618 (2) Å, θ = 176.72 (19)° and φ = 290 (3)°. The phenyl (*B*: C11–C16; *C*: C21–C26; *D*: C27–C32) rings make dihedral angles of 78.23 (10), 83.20 (11) and 82.09 (10)°, respectively, with the mean plane of the central cyclo­hexane ring. The dihedral angles between the phenyl rings are *B*/*C* = 21.88 (10)°, *B*/*D* = 21.88 (19)° and *C*/*D* = 73.64 (10)°. The C1—C7—C10—C11, C1—C7—C10—O2, C1—C7—C8—C9 and C1—C7—C8—O1 torsion angles are −157.13 (16), 27.9 (2), −73.6 (2) and 106.7 (2)°. The phenyl, benzoyl, hy­droxy, cyano C2—C17≡N1 and 1,3-dioxo-1-phenyl­butan-2-yl substituents all occupy equatorial sites, so that the cyano C2—C18≡N2 substituent necessarily occupies an axial site. There are five stereogenic centres and the chirality about the C1, C3, C4, C5 and C7 atoms are *S*, *R*, *R*, *S* and *R*, respectively. The values of the geometric parameters of the title compound are normal and compatible with those of related compounds compiled in the *Database survey* section (§5).

## 3. Supra­molecular features

**Figure 2:**
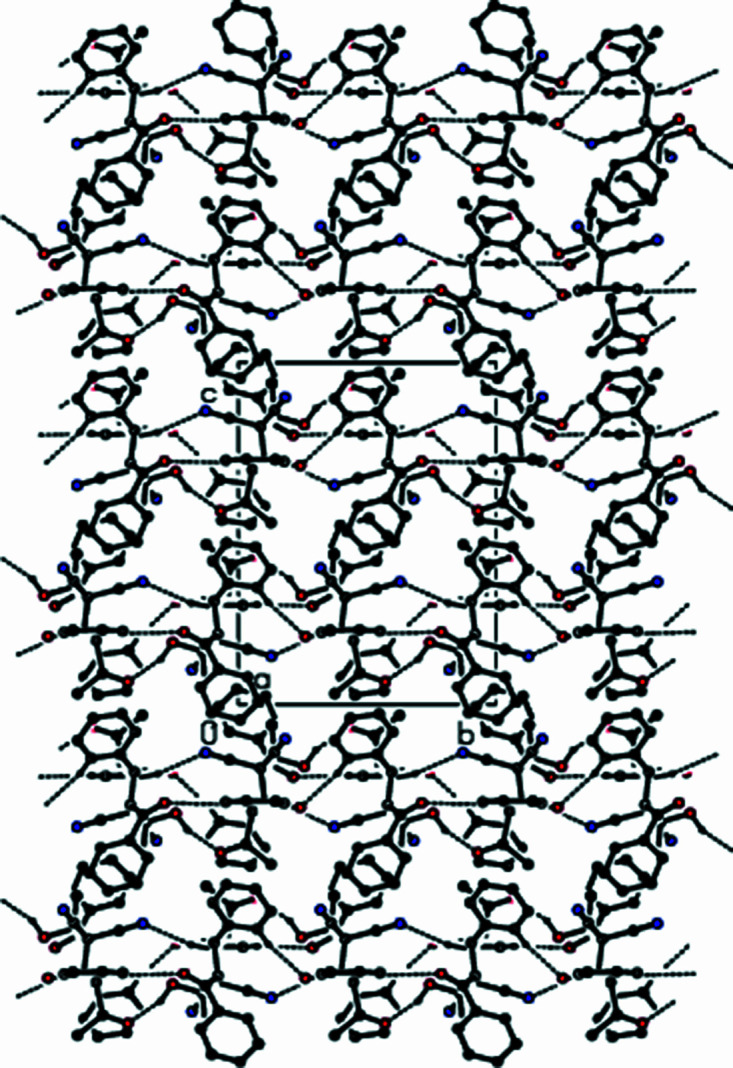
A view of the mol­ecular packing down [100], showing O—H⋯O, C—H⋯O and C—H⋯N hydrogen bonds as dashed lines.

**Figure 5:**
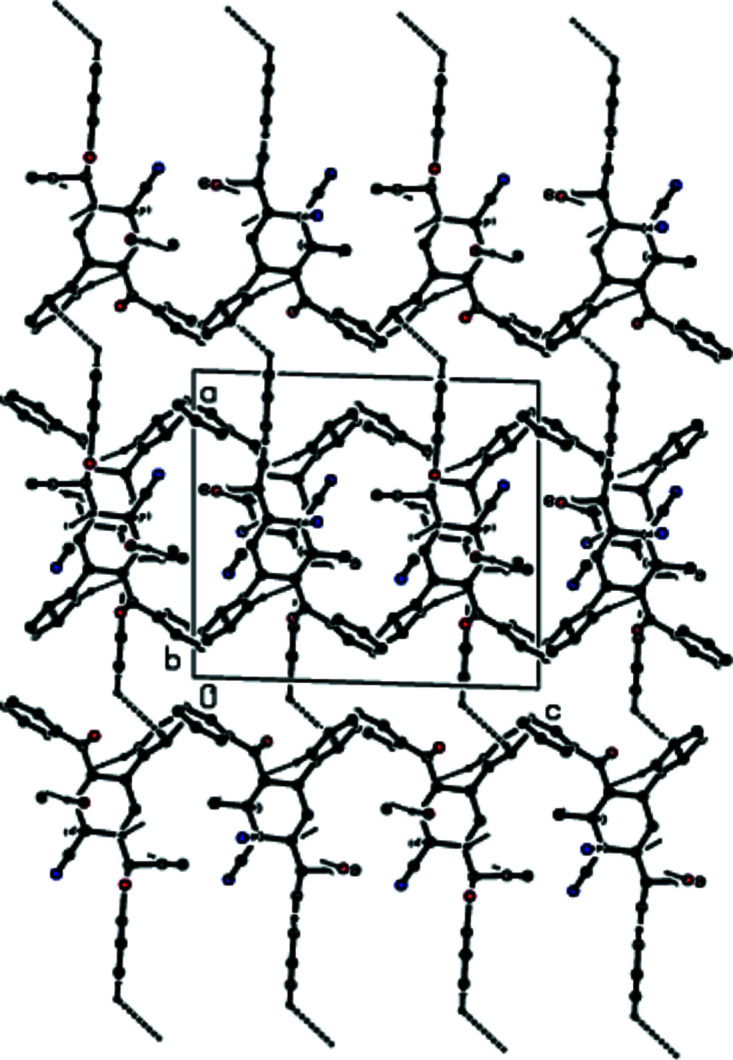
The crystal packing viewed down [010], showing O—H⋯O, C—H⋯O, C—H⋯N hydrogen bonds and C—H⋯π inter­actions.

In the crystal, O—H⋯O hydrogen bonds of medium strength, and weaker C—H⋯O and C—H⋯N inter­actions link adjacent mol­ecules, forming layers extending parallel to (100) (Table 1 ▸ and Figs. 2 ▸–4 ▸ ▸). These layers are connected by weak C—H⋯π inter­actions and van der Waals inter­actions (Table 1 ▸ and Fig. 5 ▸).

## 4. Hirshfeld surface analysis

**Figure 6:**
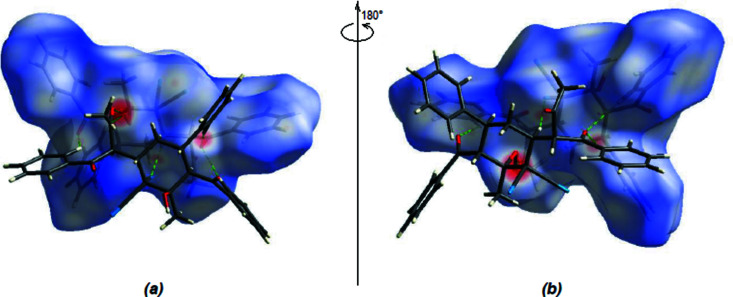
(*a*) Front and (*b*) back sides of the 3D Hirshfeld surface of the title compound plotted over *d*_norm_ in the range from −0.5877 to +1.7202 a.u.

A Hirshfeld surface for the title compound and its associated two-dimensional fingerprint plots were analyzed and calculated using *CrystalExplorer* (Version 17.5; Turner *et al.*, 2017 ▸). Hirshfeld surfaces allow for the display of inter­molecular inter­actions by using distinct colours and intensities to indicate short and long contacts, as well as the relative strengths of the inter­actions. The three-dimensional (3D) Hirshfeld surface of the title compound plotted over *d*_norm_ in the range from −0.5877 to +1.7202 a.u. is shown in Fig. 6 ▸. As discussed above, the O3—H3⋯O1 inter­actions play a key role in the mol­ecular packing of the title compound.

**Figure 7:**
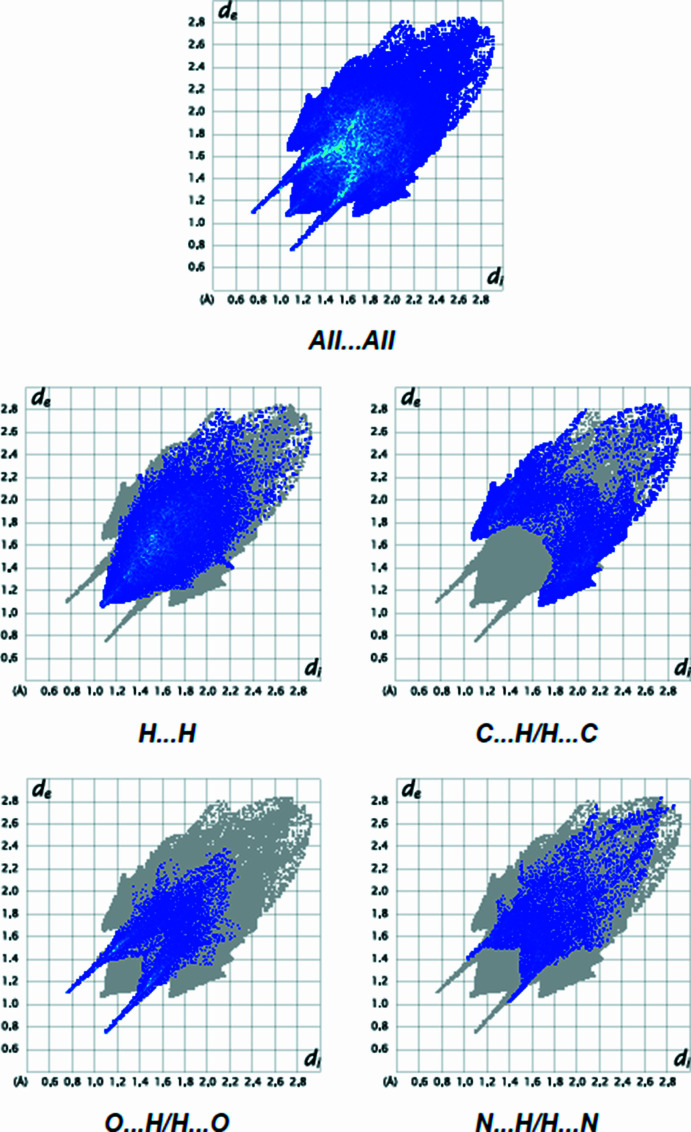
A view of the 2D fingerprint plots for the title compound, showing (*a*) all inter­actions, and delineated into (*b*) H⋯H, (*c*) C⋯H/H⋯C, (*d*) O⋯H/H⋯O and (*e*) N⋯H/H⋯N inter­actions. The *d*_i_ and *d*_e_ values are the closest inter­nal and external distances (in Å) from given points on the Hirshfeld surface.

The overall two-dimensional (2D) fingerprint plot [Fig. 7 ▸(*a*)] and those delineated into H⋯H (41.2% contribution), C⋯H/H⋯C (20.3%), O⋯H/H⋯O (17.8%) and N⋯H/H⋯N (10.6%) contacts are illustrated in Figs. 7 ▸(*b*)–(*e*), respectively. The other minor contributions to the Hirshfeld surface are from N⋯C/C⋯N (1.0%), C⋯C (0.9%), O⋯N/N⋯O (0.8%) and O⋯C/C⋯O (0.8%) contacts. The large number of H⋯H, C⋯H/H⋯C, O⋯H/H⋯O and N⋯H/H⋯N inter­actions suggest that van der Waals inter­actions and hydrogen bonding play major roles in the crystal packing. Various inter­atomic contacts are compiled in Table 2 ▸.

## 5. Database survey

A search of the Cambridge Structural Database (CSD, Version 5.42, update of September 2021; Groom *et al.*, 2016 ▸) for the 2-hydroxy-2-methylcyclohexane-1,1-dicarbonitrile moiety revealed five structures closely related to the title compound: 3-cyano-4-hy­droxy-2-(4-methyl­phen­yl)-6-oxo-*N*-phenyl-4-(thio­phen-2-yl)cyclo­hexane-1-car­boxamide hydrate (CSD refcode UPOMOE; Naghiyev *et al.*, 2021 ▸), (2*RS*,3*SR*,4*RS*,6*SR*)-3-benzoyl-4-hy­droxy-2,4,6-tri­phenyl­cyclo­hexane-1,1-dicarbo­nitrile (MEHMOC01; Rodríguez *et al.*, 2008 ▸), 3-(4-fluoro­benzo­yl)-4-(4-fluoro­phen­yl)-4-hy­droxy-2,6-di­phenyl­cyclo­hexane-1,1-dicarbo­nitrile (SODHAW; Narayana *et al.*, 2014 ▸), 5-cyano-2-hy­droxy-2-methyl-*N*-phenyl-4-(pyridin-4-yl)-6-(thio­phen-2-yl)-3,4-di­hydro-2*H*-pyran-3-carboxamide (JUPHUA; Naghiyev *et al.*, 2020 ▸) and 5-cyano-2-hy­droxy-2-methyl-6-oxo-*N*-phenyl-4-(thio­phen-2-yl)piperi­dine-3-car­box­amide methanol solvate (JUPJOW; Naghiyev *et al.*, 2020 ▸).

In the crystal of UPOMOE, the central cyclo­hexane ring adopts a chair conformation. Mol­ecules are linked by N—H⋯O, C—H⋯O and C—H⋯N hydrogen bonds, forming layers parallel to (100), which inter­act *via* the van der Waals forces between them.

In the crystal of MEHMOC01, the mol­ecules are linked into complex sheets by two C—H⋯O hydrogen bonds and three C—H⋯N hydrogen bonds.

In the crystal of SODHAW, mol­ecules are linked *via* pairs of O—H⋯N hydrogen bonds, forming inversion dimers. The dimers are linked *via* C—H⋯N and C—H⋯O hydrogen bonds, forming chains parallel to [001]. C—H⋯F hydrogen bonds link the chains into sheets lying parallel to (100).

In JUPHUA, the crystal structure is stabilized by an extensive hydrogen-bonding network defined by N—H⋯N, O—H⋯N and C—H⋯O inter­actions with graph-set motifs *C*(9), *C*(8), 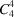 (32) and 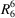 (48), with base vectors [100], [011] and [110] for the 3D network.

In JUPJOW, the crystal structure is also stabilized by an extensive hydrogen-bonding network of N—H⋯O, O—H⋯O and O—H⋯N inter­actions, where the methanol mol­ecule participate with neighbouring mol­ecules with graph-set motifs *C*(4), 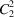 (10), 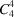 (28), 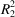 (8) and 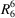 (36), with base vectors [010], [100] and [001] for the 3D network. For JUPHUA and JUPJOW, another non-covalent weak inter­action is also observed, specifically a chalcogen⋯π interaction (*ca* 3.6 Å) in JUPHUA between the thiophenyl sulfur fragment and the phenyl ring and a hydrogen⋯π interaction (*ca* 3.2 Å) in JUPJOW between the methyl group on the piperidone ring and the phenyl ring.

## 6. Synthesis and crystallization

To a solution of 2-(3-phenyl­allyl­idene)malono­nitrile (0.92 g, 5 mmol) and benzoyl­acetone (1.68 g, 10 mmol) in benzene (25 ml), 3–4 drops of 1-methyl­piperazine were added and the mixture was stirred for 10 min and kept at room temperature for 72 h. Benzene (15 ml) was then removed from the reaction mixture by distillation, which was left overnight. The crystals which formed were separated by filtration and recrystallized from an ethanol–water (1:1 *v*/*v*) solution (yield 41%; m.p. 514–515 K).

^1^H NMR (300 MHz, DMSO-*d*_6_, ppm): δ 1.74 (*s*, 3H, CH_3_), 2.01 (*t*, 2H, CH_2_), 2.12 (*s*, 3H, COCH_3_), 3.47 (*d*–*d*, 1H, CH), 3.52 (*s*, 1H, OH), 4.08 (*m*, 1H, CH), 4.62 (*d*, 1H, CH), 4.86 (*d*, 1H, CH), 7.12–7.78 (*m*, 15H, 15Ar-H). ^13^C NMR (75 MHz, DMSO-*d*_6_, ppm): δ 24.28 (CH_3_), 30.36 (COCH_3_), 34.42 (CH_2_), 39.41 (CH), 45.49 (CH), 56.46 (C_*tert*_), 57.01 (CH), 60.85 (CH), 81.92 (O—C_*tert*_), 111.37 (CN), 111.81 (CN), 125.94 (CH_arom_), 127.22 (2CH_arom_), 127.86 (2CH_arom_), 128.90 (2CH_arom_), 128.98 (2CH_arom_), 129.31 (2CH_arom_), 130.35 (2CH_arom_), 132.52 (CH_arom_), 133.85 (CH_arom_), 135.44 (C_arom_), 138.49 (C_arom_), 141.22 (C_arom_), 194.97 (C=O), 195.93 (C=O), 200.21 (C=O).

## 7. Refinement details

Crystal data, data collection and structure refinement details are summarized in Table 3 ▸. Due to large differences between calculated and observed intensities, about 40 reflections were omitted from the refinement. The H atom of the OH group was located in a difference map and its positional parameters were allowed to refine freely [O3—H3 = 0.93 (3) Å], with *U*_iso_(H) = 1.5*U*_eq_(O). All H atoms bound to C atoms were positioned geometrically and refined as riding, with C—H = 0.95 (aromatic), 0.99 (methyl­ene), 1.00 (methine) and 0.98 Å (meth­yl), with *U*_iso_(H) = 1.5*U*_eq_(C) for methyl H atoms and 1.2*U*_eq_(C) for the others.

## Supplementary Material

Crystal structure: contains datablock(s) I. DOI: 10.1107/S2056989022004777/wm5643sup1.cif

Structure factors: contains datablock(s) I. DOI: 10.1107/S2056989022004777/wm5643Isup2.hkl

Supporting information file. DOI: 10.1107/S2056989022004777/wm5643Isup3.cml

CCDC reference: 2170547

Additional supporting information:  crystallographic information; 3D view; checkCIF report

## Figures and Tables

**Figure 3:**
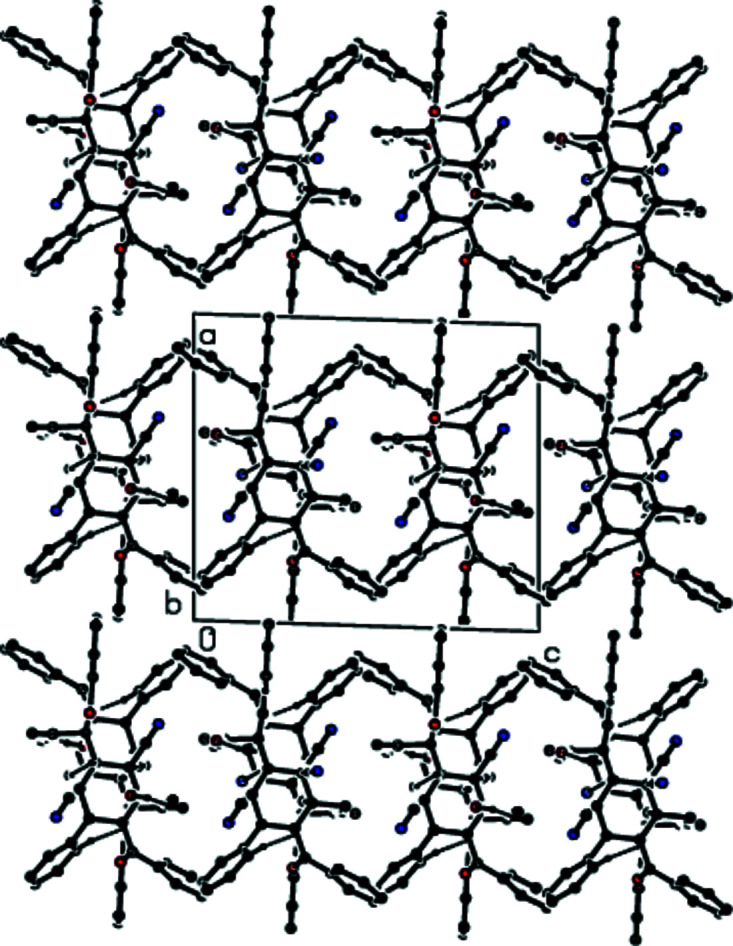
A view of the mol­ecular packing down [010]. Inter­molecular inter­actions are depicted as in Fig. 2 ▸.

**Figure 4:**
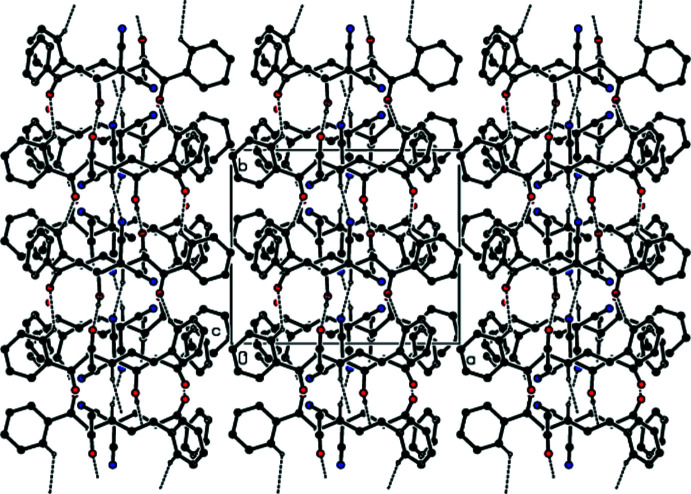
A view of the mol­ecular packing down [001]. Inter­molecular inter­actions are depicted as in Fig. 2 ▸.

**Table 1:** Hydrogen-bond geometry (Å, °) *Cg*4 is the centroid of the C27–C32 phenyl ring.

*D*—H⋯*A*	*D*—H	H⋯*A*	*D*⋯*A*	*D*—H⋯*A*
O3—H3⋯O1^i^	0.94 (3)	1.89 (3)	2.787 (2)	159 (3)
C1—H1⋯N1^i^	1.00	2.50	3.466 (3)	163
C12—H12⋯O4^ii^	0.95	2.58	3.242 (3)	127
C28—H28⋯O2^ii^	0.95	2.40	3.319 (2)	164
C14—H14⋯*Cg*4^iii^	0.95	2.80	3.475 (2)	129

Symmetry codes: (i) 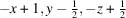 ; (ii) 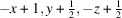 ; (iii) 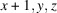 .

**Table 2:** Summary of short inter­atomic contacts (Å) in the title compound

Contact	Distance	Symmetry operation
O1⋯H3	1.89	1 − *x*, 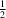 + *y*, 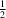 − *z*
H9*A*⋯H6*A*	2.39	1 − *x*, 1 − *y*, − *z*
O4⋯H15	2.73	−1 + *x*, *y*, *z*
H19*B*⋯N1	2.77	1 − *x*, 1 − *y*, 1 − *z*
H26⋯H31	2.37	*x*, 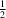 − *y*, 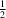 + *z*
C25⋯C24	3.367	−*x*, 1 − *y*, 1 − *z*
H29⋯H23	2.41	*x*, 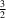 − *y*, − 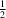 + *z*
H13⋯H15	2.36	2 − *x*, 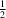 + *y*, 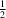 − *z*

**Table 3:** Experimental details

Crystal data	
Chemical formula	C_32_H_28_N_2_O_4_
*M*_r_	504.56
Crystal system, space group	Monoclinic, *P*2_1_/*c*
Temperature (K)	100
*a*, *b*, *c* (Å)	13.9798 (3), 11.8411 (2), 15.7406 (3)
β (°)	91.901 (2)
*V* (Å^3^)	2604.21 (9)
*Z*	4
Radiation type	Cu *K*α
μ (mm^−1^)	0.69
Crystal size (mm)	0.09 × 0.06 × 0.06

Data collection	
Diffractometer	Rigaku XtaLAB Synergy Dualflex HyPix
Absorption correction	Multi-scan (*CrysAlis PRO*; Rigaku OD, 2021 ▸)
*T*_min_, *T*_max_	0.933, 0.949
No. of measured, independent and observed [*I* > 2σ(*I*)] reflections	77914, 5618, 5497
*R*_int_	0.110
(sin θ/λ)_max_ (Å^−1^)	0.639

Refinement	
*R*[*F* ^2^ > 2σ(*F* ^2^)], *wR*(*F* ^2^), *S*	0.074, 0.187, 1.11
No. of reflections	5618
No. of parameters	348
H-atom treatment	H atoms treated by a mixture of independent and constrained refinement
Δρ_max_, Δρ_min_ (e Å^−3^)	0.54, −0.33

Computer programs: *CrysAlis PRO* (Rigaku OD, 2021 ▸), *SHELXT* (Sheldrick, 2015*a* ▸), *SHELXL* (Sheldrick, 2015*b* ▸), *ORTEP-3 for Windows* (Farrugia, 2012 ▸) and *PLATON* (Spek, 2020 ▸).

## supplementary crystallographic information

### Crystal data

**Table:**

C_32_H_28_N_2_O_4_	*F*(000) = 1064
*M**_r_* = 504.56	*D*_x_ = 1.287 Mg m^−^^3^
Monoclinic, *P*2_1_/*c*	Cu *K*α radiation, λ = 1.54184 Å
*a* = 13.9798 (3) Å	Cell parameters from 52541 reflections
*b* = 11.8411 (2) Å	θ = 3.1–79.4°
*c* = 15.7406 (3) Å	µ = 0.69 mm^−^^1^
β = 91.901 (2)°	*T* = 100 K
*V* = 2604.21 (9) Å^3^	Prism, colourless
*Z* = 4	0.09 × 0.06 × 0.06 mm

### Data collection

**Table:**

Rigaku XtaLAB Synergy Dualflex HyPix diffractometer	5497 reflections with *I* > 2σ(*I*)
Radiation source: micro-focus sealed X-ray tube	*R*_int_ = 0.110
φ and ω scans	θ_max_ = 80.3°, θ_min_ = 4.7°
Absorption correction: multi-scan (CrysAlis PRO; Rigaku OD, 2021)	*h* = −17→17
*T*_min_ = 0.933, *T*_max_ = 0.949	*k* = −14→12
77914 measured reflections	*l* = −20→20
5618 independent reflections	

### Refinement

**Table:**

Refinement on *F*^2^	Primary atom site location: difference Fourier map
Least-squares matrix: full	Secondary atom site location: difference Fourier map
*R*[*F*^2^ > 2σ(*F*^2^)] = 0.074	Hydrogen site location: mixed
*wR*(*F*^2^) = 0.187	H atoms treated by a mixture of independent and constrained refinement
*S* = 1.11	*w* = 1/[σ^2^(*F*_o_^2^) + (0.0869*P*)^2^ + 2.2708*P*] where *P* = (*F*_o_^2^ + 2*F*_c_^2^)/3
5618 reflections	(Δ/σ)_max_ < 0.001
348 parameters	Δρ_max_ = 0.54 e Å^−^^3^
0 restraints	Δρ_min_ = −0.33 e Å^−^^3^

### Special details

**Table:**

Geometry. All esds (except the esd in the dihedral angle between two l.s. planes) are estimated using the full covariance matrix. The cell esds are taken into account individually in the estimation of esds in distances, angles and torsion angles; correlations between esds in cell parameters are only used when they are defined by crystal symmetry. An approximate (isotropic) treatment of cell esds is used for estimating esds involving l.s. planes.

### Fractional atomic coordinates and isotropic or equivalent isotropic displacement parameters (Å^2^)

**Table:**

	*x*	*y*	*z*	*U*_iso_*/*U*_eq_	
O1	0.60930 (11)	0.56945 (13)	0.06669 (10)	0.0327 (3)	
O2	0.68557 (10)	0.25976 (12)	0.19898 (9)	0.0290 (3)	
O3	0.42148 (11)	0.24428 (13)	0.31883 (10)	0.0324 (3)	
H3	0.415 (2)	0.199 (3)	0.367 (2)	0.049*	
O4	0.20303 (11)	0.28810 (13)	0.28805 (10)	0.0351 (4)	
N1	0.52004 (13)	0.62769 (17)	0.35886 (12)	0.0344 (4)	
N2	0.65726 (13)	0.31942 (18)	0.39833 (12)	0.0368 (4)	
C1	0.52388 (13)	0.39859 (16)	0.21623 (12)	0.0231 (4)	
H1	0.5225	0.3158	0.2038	0.028*	
C2	0.51310 (13)	0.41460 (17)	0.31391 (12)	0.0240 (4)	
C3	0.41622 (13)	0.35964 (18)	0.34400 (12)	0.0266 (4)	
C4	0.33213 (13)	0.42000 (17)	0.29384 (12)	0.0237 (4)	
H4	0.3342	0.5025	0.3074	0.028*	
C5	0.34164 (13)	0.40464 (16)	0.19736 (12)	0.0236 (4)	
H5	0.3408	0.3219	0.1846	0.028*	
C6	0.43762 (13)	0.45324 (17)	0.16959 (12)	0.0244 (4)	
H6A	0.4432	0.4417	0.1077	0.029*	
H6B	0.4386	0.5356	0.1805	0.029*	
C7	0.62107 (13)	0.44637 (16)	0.18714 (12)	0.0237 (4)	
H7	0.6368	0.5172	0.2193	0.028*	
C8	0.61386 (13)	0.47219 (17)	0.09111 (12)	0.0261 (4)	
C9	0.61333 (15)	0.3741 (2)	0.03178 (13)	0.0320 (4)	
H9A	0.5778	0.3941	−0.0208	0.048*	
H9B	0.5825	0.3095	0.0585	0.048*	
H9C	0.6793	0.3543	0.0187	0.048*	
C10	0.70293 (13)	0.36013 (16)	0.20056 (12)	0.0235 (4)	
C11	0.80373 (13)	0.40261 (17)	0.20804 (12)	0.0255 (4)	
C12	0.82669 (14)	0.51657 (19)	0.20982 (14)	0.0308 (4)	
H12	0.7773	0.5717	0.2078	0.037*	
C13	0.92204 (15)	0.5500 (2)	0.21452 (15)	0.0352 (5)	
H13	0.9376	0.6281	0.2152	0.042*	
C14	0.99465 (15)	0.4702 (2)	0.21823 (15)	0.0364 (5)	
H14	1.0597	0.4935	0.2220	0.044*	
C15	0.97192 (15)	0.3563 (2)	0.21636 (17)	0.0403 (6)	
H15	1.0215	0.3015	0.2188	0.048*	
C16	0.87744 (15)	0.32241 (19)	0.21094 (15)	0.0346 (5)	
H16	0.8623	0.2442	0.2092	0.041*	
C17	0.51666 (13)	0.53547 (18)	0.33812 (12)	0.0257 (4)	
C18	0.59353 (14)	0.35950 (18)	0.36132 (12)	0.0269 (4)	
C19	0.40934 (15)	0.3705 (2)	0.43962 (13)	0.0343 (5)	
H19A	0.4054	0.4505	0.4550	0.051*	
H19B	0.4662	0.3368	0.4675	0.051*	
H19C	0.3520	0.3311	0.4581	0.051*	
C20	0.23709 (13)	0.37142 (17)	0.32275 (12)	0.0254 (4)	
C21	0.19117 (14)	0.42334 (19)	0.39784 (13)	0.0301 (4)	
C22	0.20292 (16)	0.5357 (2)	0.41997 (16)	0.0403 (5)	
H22	0.2385	0.5844	0.3849	0.048*	
C23	0.16292 (19)	0.5777 (3)	0.4932 (2)	0.0582 (8)	
H23	0.1697	0.6553	0.5074	0.070*	
C24	0.1136 (2)	0.5067 (4)	0.54509 (18)	0.0669 (10)	
H24	0.0878	0.5351	0.5959	0.080*	
C25	0.1012 (2)	0.3952 (4)	0.5241 (2)	0.0663 (10)	
H25	0.0675	0.3465	0.5606	0.080*	
C26	0.13807 (17)	0.3531 (3)	0.44923 (17)	0.0457 (6)	
H26	0.1270	0.2767	0.4333	0.055*	
C27	0.26091 (13)	0.46041 (17)	0.14557 (12)	0.0243 (4)	
C28	0.23681 (13)	0.57302 (17)	0.15741 (13)	0.0261 (4)	
H28	0.2681	0.6150	0.2016	0.031*	
C29	0.16717 (14)	0.62547 (19)	0.10523 (13)	0.0297 (4)	
H29	0.1507	0.7022	0.1147	0.036*	
C30	0.12222 (14)	0.5654 (2)	0.03973 (14)	0.0330 (5)	
H30	0.0756	0.6011	0.0035	0.040*	
C31	0.14567 (15)	0.4533 (2)	0.02747 (14)	0.0346 (5)	
H31	0.1149	0.4119	−0.0173	0.042*	
C32	0.21395 (15)	0.40056 (19)	0.08018 (13)	0.0301 (4)	
H32	0.2287	0.3232	0.0716	0.036*	

### Atomic displacement parameters (Å^2^)

**Table:**

	*U*^11^	*U*^22^	*U*^33^	*U*^12^	*U*^13^	*U*^23^
O1	0.0331 (8)	0.0336 (8)	0.0315 (8)	0.0030 (6)	0.0039 (6)	0.0075 (6)
O2	0.0234 (7)	0.0273 (7)	0.0364 (8)	0.0000 (5)	0.0006 (5)	0.0033 (6)
O3	0.0323 (8)	0.0267 (7)	0.0385 (8)	−0.0003 (6)	0.0031 (6)	0.0060 (6)
O4	0.0280 (7)	0.0334 (8)	0.0444 (9)	−0.0061 (6)	0.0048 (6)	−0.0021 (7)
N1	0.0320 (9)	0.0354 (10)	0.0355 (9)	0.0023 (7)	−0.0010 (7)	−0.0007 (8)
N2	0.0290 (9)	0.0458 (11)	0.0353 (10)	0.0044 (8)	−0.0025 (7)	0.0073 (8)
C1	0.0188 (8)	0.0272 (9)	0.0234 (9)	−0.0002 (7)	0.0000 (6)	0.0028 (7)
C2	0.0198 (8)	0.0288 (10)	0.0233 (9)	0.0014 (7)	−0.0017 (7)	0.0021 (7)
C3	0.0215 (9)	0.0329 (10)	0.0255 (9)	0.0000 (7)	0.0005 (7)	0.0063 (8)
C4	0.0197 (8)	0.0279 (9)	0.0236 (9)	0.0002 (7)	0.0009 (7)	0.0011 (7)
C5	0.0195 (8)	0.0276 (9)	0.0236 (9)	−0.0009 (7)	−0.0003 (6)	0.0014 (7)
C6	0.0207 (8)	0.0319 (10)	0.0204 (8)	0.0006 (7)	0.0006 (6)	0.0034 (7)
C7	0.0193 (8)	0.0270 (9)	0.0248 (9)	0.0007 (7)	0.0002 (6)	0.0028 (7)
C8	0.0166 (8)	0.0338 (10)	0.0280 (9)	0.0010 (7)	0.0026 (6)	0.0046 (8)
C9	0.0304 (10)	0.0407 (12)	0.0250 (9)	0.0034 (8)	0.0017 (8)	0.0022 (8)
C10	0.0209 (8)	0.0272 (9)	0.0225 (8)	0.0020 (7)	0.0011 (6)	0.0030 (7)
C11	0.0199 (8)	0.0318 (10)	0.0248 (9)	0.0022 (7)	0.0009 (7)	0.0029 (7)
C12	0.0230 (9)	0.0331 (11)	0.0361 (11)	0.0012 (8)	−0.0006 (7)	0.0009 (8)
C13	0.0245 (10)	0.0347 (11)	0.0462 (12)	−0.0045 (8)	−0.0024 (8)	−0.0007 (9)
C14	0.0193 (9)	0.0457 (13)	0.0441 (12)	−0.0019 (8)	−0.0011 (8)	0.0112 (10)
C15	0.0216 (10)	0.0410 (13)	0.0585 (15)	0.0064 (8)	0.0042 (9)	0.0166 (11)
C16	0.0228 (9)	0.0328 (11)	0.0482 (13)	0.0028 (8)	0.0027 (8)	0.0112 (9)
C17	0.0196 (8)	0.0335 (11)	0.0238 (9)	0.0016 (7)	−0.0016 (6)	0.0025 (7)
C18	0.0235 (9)	0.0335 (10)	0.0235 (9)	0.0007 (7)	0.0006 (7)	0.0045 (8)
C19	0.0251 (10)	0.0536 (13)	0.0242 (10)	0.0017 (9)	0.0007 (7)	0.0010 (9)
C20	0.0226 (9)	0.0269 (9)	0.0266 (9)	0.0015 (7)	0.0002 (7)	0.0047 (7)
C21	0.0187 (8)	0.0427 (12)	0.0289 (10)	0.0045 (8)	0.0020 (7)	0.0038 (8)
C22	0.0237 (10)	0.0520 (14)	0.0453 (13)	0.0038 (9)	0.0017 (9)	−0.0153 (11)
C23	0.0311 (12)	0.088 (2)	0.0552 (17)	0.0090 (13)	0.0003 (11)	−0.0338 (16)
C24	0.0444 (15)	0.121 (3)	0.0360 (13)	0.0343 (18)	0.0033 (11)	−0.0123 (16)
C25	0.0412 (15)	0.112 (3)	0.0469 (16)	0.0273 (16)	0.0208 (12)	0.0340 (18)
C26	0.0336 (12)	0.0575 (16)	0.0470 (14)	0.0113 (10)	0.0145 (10)	0.0213 (12)
C27	0.0181 (8)	0.0312 (10)	0.0236 (9)	−0.0027 (7)	0.0015 (6)	0.0025 (7)
C28	0.0222 (9)	0.0288 (10)	0.0271 (9)	−0.0025 (7)	−0.0007 (7)	0.0017 (7)
C29	0.0236 (9)	0.0322 (10)	0.0333 (10)	0.0007 (8)	0.0002 (7)	0.0045 (8)
C30	0.0225 (9)	0.0446 (12)	0.0315 (10)	0.0006 (8)	−0.0037 (7)	0.0076 (9)
C31	0.0267 (10)	0.0446 (12)	0.0321 (10)	−0.0014 (9)	−0.0076 (8)	−0.0044 (9)
C32	0.0261 (9)	0.0340 (11)	0.0300 (10)	0.0012 (8)	−0.0023 (8)	−0.0039 (8)

### Geometric parameters (Å, º)

**Table:**

O1—C8	1.215 (3)	C12—H12	0.9500
O2—C10	1.213 (2)	C13—C14	1.387 (3)
O3—C3	1.425 (3)	C13—H13	0.9500
O3—H3	0.93 (3)	C14—C15	1.386 (4)
O4—C20	1.217 (3)	C14—H14	0.9500
N1—C17	1.140 (3)	C15—C16	1.380 (3)
N2—C18	1.151 (3)	C15—H15	0.9500
C1—C6	1.534 (2)	C16—H16	0.9500
C1—C7	1.555 (2)	C19—H19A	0.9800
C1—C2	1.561 (3)	C19—H19B	0.9800
C1—H1	1.0000	C19—H19C	0.9800
C2—C18	1.480 (3)	C20—C21	1.496 (3)
C2—C17	1.481 (3)	C21—C22	1.384 (3)
C2—C3	1.589 (3)	C21—C26	1.392 (3)
C3—C19	1.517 (3)	C22—C23	1.390 (4)
C3—C4	1.567 (3)	C22—H22	0.9500
C4—C20	1.531 (3)	C23—C24	1.374 (6)
C4—C5	1.540 (3)	C23—H23	0.9500
C4—H4	1.0000	C24—C25	1.370 (6)
C5—C27	1.521 (3)	C24—H24	0.9500
C5—C6	1.537 (3)	C25—C26	1.393 (4)
C5—H5	1.0000	C25—H25	0.9500
C6—H6A	0.9900	C26—H26	0.9500
C6—H6B	0.9900	C27—C28	1.389 (3)
C7—C8	1.542 (3)	C27—C32	1.396 (3)
C7—C10	1.543 (3)	C28—C29	1.398 (3)
C7—H7	1.0000	C28—H28	0.9500
C8—C9	1.490 (3)	C29—C30	1.386 (3)
C9—H9A	0.9800	C29—H29	0.9500
C9—H9B	0.9800	C30—C31	1.383 (3)
C9—H9C	0.9800	C30—H30	0.9500
C10—C11	1.497 (3)	C31—C32	1.392 (3)
C11—C12	1.387 (3)	C31—H31	0.9500
C11—C16	1.401 (3)	C32—H32	0.9500
C12—C13	1.390 (3)		
			
C3—O3—H3	108 (2)	C13—C12—H12	120.0
C6—C1—C7	112.72 (15)	C14—C13—C12	120.5 (2)
C6—C1—C2	108.66 (15)	C14—C13—H13	119.8
C7—C1—C2	111.08 (15)	C12—C13—H13	119.8
C6—C1—H1	108.1	C15—C14—C13	119.7 (2)
C7—C1—H1	108.1	C15—C14—H14	120.2
C2—C1—H1	108.1	C13—C14—H14	120.2
C18—C2—C17	106.12 (16)	C16—C15—C14	120.2 (2)
C18—C2—C1	110.26 (16)	C16—C15—H15	119.9
C17—C2—C1	111.54 (16)	C14—C15—H15	119.9
C18—C2—C3	108.05 (15)	C15—C16—C11	120.4 (2)
C17—C2—C3	109.92 (16)	C15—C16—H16	119.8
C1—C2—C3	110.78 (15)	C11—C16—H16	119.8
O3—C3—C19	111.25 (17)	N1—C17—C2	178.2 (2)
O3—C3—C4	110.00 (16)	N2—C18—C2	178.2 (2)
C19—C3—C4	112.97 (17)	C3—C19—H19A	109.5
O3—C3—C2	104.92 (15)	C3—C19—H19B	109.5
C19—C3—C2	110.11 (16)	H19A—C19—H19B	109.5
C4—C3—C2	107.20 (15)	C3—C19—H19C	109.5
C20—C4—C5	110.67 (15)	H19A—C19—H19C	109.5
C20—C4—C3	108.84 (15)	H19B—C19—H19C	109.5
C5—C4—C3	110.81 (15)	O4—C20—C21	121.07 (18)
C20—C4—H4	108.8	O4—C20—C4	120.11 (18)
C5—C4—H4	108.8	C21—C20—C4	118.68 (17)
C3—C4—H4	108.8	C22—C21—C26	119.3 (2)
C27—C5—C6	108.92 (15)	C22—C21—C20	122.9 (2)
C27—C5—C4	112.99 (15)	C26—C21—C20	117.7 (2)
C6—C5—C4	109.95 (15)	C21—C22—C23	120.4 (3)
C27—C5—H5	108.3	C21—C22—H22	119.8
C6—C5—H5	108.3	C23—C22—H22	119.8
C4—C5—H5	108.3	C24—C23—C22	119.8 (3)
C1—C6—C5	112.69 (15)	C24—C23—H23	120.1
C1—C6—H6A	109.1	C22—C23—H23	120.1
C5—C6—H6A	109.1	C25—C24—C23	120.5 (3)
C1—C6—H6B	109.1	C25—C24—H24	119.7
C5—C6—H6B	109.1	C23—C24—H24	119.7
H6A—C6—H6B	107.8	C24—C25—C26	120.1 (3)
C8—C7—C10	106.82 (15)	C24—C25—H25	119.9
C8—C7—C1	109.36 (15)	C26—C25—H25	119.9
C10—C7—C1	111.73 (15)	C21—C26—C25	119.8 (3)
C8—C7—H7	109.6	C21—C26—H26	120.1
C10—C7—H7	109.6	C25—C26—H26	120.1
C1—C7—H7	109.6	C28—C27—C32	118.43 (18)
O1—C8—C9	122.77 (18)	C28—C27—C5	121.58 (17)
O1—C8—C7	119.93 (18)	C32—C27—C5	119.82 (18)
C9—C8—C7	117.30 (17)	C27—C28—C29	120.97 (18)
C8—C9—H9A	109.5	C27—C28—H28	119.5
C8—C9—H9B	109.5	C29—C28—H28	119.5
H9A—C9—H9B	109.5	C30—C29—C28	119.9 (2)
C8—C9—H9C	109.5	C30—C29—H29	120.0
H9A—C9—H9C	109.5	C28—C29—H29	120.0
H9B—C9—H9C	109.5	C31—C30—C29	119.51 (19)
O2—C10—C11	121.19 (17)	C31—C30—H30	120.2
O2—C10—C7	119.91 (17)	C29—C30—H30	120.2
C11—C10—C7	118.70 (16)	C30—C31—C32	120.6 (2)
C12—C11—C16	119.29 (18)	C30—C31—H31	119.7
C12—C11—C10	123.02 (17)	C32—C31—H31	119.7
C16—C11—C10	117.66 (18)	C31—C32—C27	120.5 (2)
C11—C12—C13	119.9 (2)	C31—C32—H32	119.7
C11—C12—H12	120.0	C27—C32—H32	119.7
			
C6—C1—C2—C18	178.10 (16)	O2—C10—C11—C12	179.81 (19)
C7—C1—C2—C18	−57.3 (2)	C7—C10—C11—C12	4.9 (3)
C6—C1—C2—C17	−64.26 (19)	O2—C10—C11—C16	1.6 (3)
C7—C1—C2—C17	60.3 (2)	C7—C10—C11—C16	−173.24 (18)
C6—C1—C2—C3	58.5 (2)	C16—C11—C12—C13	−0.1 (3)
C7—C1—C2—C3	−176.92 (15)	C10—C11—C12—C13	−178.23 (19)
C18—C2—C3—O3	−63.67 (19)	C11—C12—C13—C14	−0.6 (4)
C17—C2—C3—O3	−179.06 (15)	C12—C13—C14—C15	0.6 (4)
C1—C2—C3—O3	57.22 (19)	C13—C14—C15—C16	−0.1 (4)
C18—C2—C3—C19	56.1 (2)	C14—C15—C16—C11	−0.6 (4)
C17—C2—C3—C19	−59.3 (2)	C12—C11—C16—C15	0.6 (3)
C1—C2—C3—C19	177.02 (17)	C10—C11—C16—C15	178.9 (2)
C18—C2—C3—C4	179.40 (16)	C5—C4—C20—O4	34.1 (2)
C17—C2—C3—C4	64.01 (19)	C3—C4—C20—O4	−87.9 (2)
C1—C2—C3—C4	−59.7 (2)	C5—C4—C20—C21	−150.15 (17)
O3—C3—C4—C20	67.89 (19)	C3—C4—C20—C21	87.8 (2)
C19—C3—C4—C20	−57.1 (2)	O4—C20—C21—C22	−155.3 (2)
C2—C3—C4—C20	−178.56 (15)	C4—C20—C21—C22	29.0 (3)
O3—C3—C4—C5	−54.0 (2)	O4—C20—C21—C26	27.6 (3)
C19—C3—C4—C5	−179.00 (17)	C4—C20—C21—C26	−148.07 (19)
C2—C3—C4—C5	59.5 (2)	C26—C21—C22—C23	0.7 (3)
C20—C4—C5—C27	58.4 (2)	C20—C21—C22—C23	−176.3 (2)
C3—C4—C5—C27	179.24 (15)	C21—C22—C23—C24	1.7 (4)
C20—C4—C5—C6	−179.68 (15)	C22—C23—C24—C25	−1.7 (4)
C3—C4—C5—C6	−58.8 (2)	C23—C24—C25—C26	−0.6 (4)
C7—C1—C6—C5	179.09 (16)	C22—C21—C26—C25	−2.9 (3)
C2—C1—C6—C5	−57.3 (2)	C20—C21—C26—C25	174.2 (2)
C27—C5—C6—C1	−177.85 (16)	C24—C25—C26—C21	2.9 (4)
C4—C5—C6—C1	57.8 (2)	C6—C5—C27—C28	−70.2 (2)
C6—C1—C7—C8	−35.8 (2)	C4—C5—C27—C28	52.3 (2)
C2—C1—C7—C8	−158.06 (16)	C6—C5—C27—C32	105.0 (2)
C6—C1—C7—C10	−153.88 (16)	C4—C5—C27—C32	−132.47 (19)
C2—C1—C7—C10	83.90 (19)	C32—C27—C28—C29	0.0 (3)
C10—C7—C8—O1	−132.18 (18)	C5—C27—C28—C29	175.32 (18)
C1—C7—C8—O1	106.7 (2)	C27—C28—C29—C30	−1.0 (3)
C10—C7—C8—C9	47.5 (2)	C28—C29—C30—C31	1.1 (3)
C1—C7—C8—C9	−73.6 (2)	C29—C30—C31—C32	−0.1 (3)
C8—C7—C10—O2	−91.6 (2)	C30—C31—C32—C27	−0.9 (3)
C1—C7—C10—O2	27.9 (2)	C28—C27—C32—C31	0.9 (3)
C8—C7—C10—C11	83.3 (2)	C5—C27—C32—C31	−174.44 (19)
C1—C7—C10—C11	−157.13 (16)		

### Hydrogen-bond geometry (Å, º)

*Cg*4 is a centroid of the C27–C32 phenyl ring.

**Table:**

*D*—H···*A*	*D*—H	H···*A*	*D*···*A*	*D*—H···*A*
O3—H3···O1^i^	0.94 (3)	1.89 (3)	2.787 (2)	159 (3)
C1—H1···O2	1.00	2.38	2.815 (2)	106
C1—H1···O3	1.00	2.48	2.855 (2)	102
C1—H1···N1^i^	1.00	2.50	3.466 (3)	163
C5—H5···O3	1.00	2.53	2.892 (2)	101
C12—H12···O4^ii^	0.95	2.58	3.242 (3)	127
C28—H28···O2^ii^	0.95	2.40	3.319 (2)	164
C14—H14···*Cg*4^iii^	0.95	2.80	3.475 (2)	129

Symmetry codes: (i) −*x*+1, *y*−1/2, −*z*+1/2; (ii) −*x*+1, *y*+1/2, −*z*+1/2; (iii) *x*+1, *y*, *z*.

## References

[bb1] Aliyeva, K. N., Maharramov, A. M., Allahverdiyev, M. A., Gurbanov, A. V. & Brito, I. (2011). *Acta Cryst.* E**67**, o2293. 10.1107/S160053681103145X PMC3200700 22058933

[bb2] Amoozadeh, A., Hosseininya, S. F. & Rahmani, S. (2018). *Res. Chem. Intermed.* **44**, 991–1011.

[bb3] Bardasov, I. N., Alekseeva, A. U., Mihailov, D. L., Ershov, O. V., Nasakin, O. E. & Tafeenko, V. A. (2014). *Tetrahedron Lett.* **55**, 2730–2733.

[bb4] Cremer, D. & Pople, J. A. (1975). *J. Am. Chem. Soc.* **97**, 1354–1358.

[bb5] Farrugia, L. J. (2012). *J. Appl. Cryst.* **45**, 849–854.

[bb6] Groom, C. R., Bruno, I. J., Lightfoot, M. P. & Ward, S. C. (2016). *Acta Cryst.* B**72**, 171–179. 10.1107/S2052520616003954 PMC4822653 27048719

[bb7] Gurbanov, A. V., Kuznetsov, M. L., Demukhamedova, S. D., Alieva, I. N., Godjaev, N. M., Zubkov, F. I., Mahmudov, K. T. & Pombeiro, A. J. L. (2020). *CrystEngComm*, **22**, 628–633.

[bb8] Kaur, N., Bhardwaj, P. & Gupta, M. (2021). *Curr. Org. Chem.* **25**, 2765–2790.

[bb9] Khalilov, A. N., Tüzün, B., Taslimi, P., Tas, A., Tuncbilek, Z. & Cakmak, N. K. (2021). *J. Mol. Liq.* **344**, 117761.

[bb10] Mamedov, I. G., Khrustalev, V. N., Akkurt, M., Novikov, A. P., Asgarova, A. R., Aliyeva, K. N. & Akobirshoeva, A. A. (2022). *Acta Cryst.* E**78**, 291–296. 10.1107/S2056989022001232 PMC8900508 35371550

[bb11] Naghiyev, F. N., Cisterna, J., Khalilov, A. N., Maharramov, A. M., Askerov, R. K., Asadov, K. A., Mamedov, I. G., Salmanli, K. S., Cárdenas, A. & Brito, I. (2020). *Molecules*, **25**, 2235. 10.3390/molecules25092235 PMC7248728 32397450

[bb12] Naghiyev, F. N., Khrustalev, V. N., Akkurt, M., Huseynov, E. Z., Khalilov, A. N., Akobirshoeva, A. A. & Mamedov, İ. G. (2021). *Acta Cryst.* E**77**, 366–371. 10.1107/S2056989021002449 PMC8025867 33936759

[bb13] Narayana, B., Sapnakumari, M., Sarojini, B. K. & Jasinski, J. P. (2014). *Acta Cryst.* E**70**, o736–o737. 10.1107/S1600536814012197 PMC4051101 24940301

[bb14] Rigaku OD (2021). *CrysAlis PRO*. Rigaku Oxford Diffraction, Tokyo, Japan.

[bb15] Rodríguez, R., Nogueras, M., Low, J. N., Cobo, J. & Glidewell, C. (2008). *Acta Cryst.* C**64**, o578–o582. 10.1107/S0108270108030515 18838780

[bb16] Safavora, A. S., Brito, I., Cisterna, J., Cardenas, A., Huseynov, E. Z., Khalilov, A. N., Naghiyev, F. N., Askerov, R. K. & Maharramov, A. M. Z. (2019). *Z. Kristallogr. New Cryst. Struct.* **234**, 1183–1185.

[bb17] Sheldrick, G. M. (2015*a*). *Acta Cryst.* A**71**, 3–8.

[bb18] Sheldrick, G. M. (2015*b*). *Acta Cryst.* C**71**, 3–8.

[bb19] Shikhaliyev, N. Q., Kuznetsov, M. L., Maharramov, A. M., Gurbanov, A. V., Ahmadova, N. E., Nenajdenko, V. G., Mahmudov, K. T. & Pombeiro, A. J. L. (2019). *CrystEngComm*, **21**, 5032–5038.

[bb20] Spek, A. L. (2020). *Acta Cryst.* E**76**, 1–11. 10.1107/S2056989019016244 PMC6944088 31921444

[bb21] Turner, M. J., MacKinnon, J. J., Wolff, S. K., Grimwood, D. J., Spackman, P. R., Jayatilaka, D. & Spackman, M. A. (2017). *CrystalExplorer 17.5*. University of Western Australia. http://hirshfeldsurface.net.

[bb22] Viswanathan, A., Kute, D., Musa, A., Konda Mani, S., Sipilä, V., Emmert-Streib, F., Zubkov, F. I., Gurbanov, A. V., Yli-Harja, O. & Kandhavelu, M. (2019). *Eur. J. Med. Chem.* **166**, 291–303. 10.1016/j.ejmech.2019.01.021 30731398

[bb23] Zubkov, F. I., Mertsalov, D. F., Zaytsev, V. P., Varlamov, A. V., Gurbanov, A. V., Dorovatovskii, P. V., Timofeeva, T. V., Khrustalev, V. N. & Mahmudov, K. T. (2018). *J. Mol. Liq.* **249**, 949–952.

